# Demographic and clinical associations with miscarriage and unsuccessful conception attempts among female Syrian refugees in Lebanon

**DOI:** 10.1186/s12884-025-07990-6

**Published:** 2025-08-12

**Authors:** Gladys Honein- AbouHaidar, Hady Naal, Asmaa El Dakdouki, Zahraa Chamseddine, Dalia Sarieddine, Hani Tamim, Tania Bosqui, Fouad Fouad, Sara Ibrahim, Zahi Abdul Sater, Shadi Saleh

**Affiliations:** 1 Hariri School of Nursing, American University of Beirut, Beirut, Lebanon; 2 Global Health Institute, American University of Beirut, Beirut, Lebanon; 3 Department of Internal Medicine, Clinical Research Institute, American University of Beirut, Beirut, Lebanon; 4 College of Medicine, Alfaisal University, Riyadh, Saudi Arabia; 5 Department of Psychology, American University of Beirut, Beirut, Lebanon; 6 Trinity Center for Global Health, Trinity College Dublin, Dublin, Ireland; 7 Department of International Public Health, Liverpool School of Tropical Medicine, Liverpool, UK; 8 Department of Health Policy and Management, American University of Beirut, Beirut, Lebanon

**Keywords:** Miscarriage, Infertility, Pregnancy Syrian refugees, Reproductive health, Sexual health, Lebanon

## Abstract

**Background:**

Female Syrian refugees in Lebanon face poor sexual reproductive health outcomes. However, associated risk and protective factors are not clearly understood. This study focuses on examining sociodemographic and clinical factors associated with miscarriage and unsuccessful conception among this population.

**Methods:**

This cross-sectional study is part of the Self-Efficacy and Knowledge Trial, which aims to enhance SRH and family planning among Female Syrian refugees in humanitarian settings. This baseline data (*n* = 485) was collected from 2 primary healthcare centers in the Bekaa in Lebanon using the PAPFAM tool during November and December 2023. Statistical analyses included chi-square tests, ANOVA, independent t-tests, and multivariate logistic regression to identify associations between sociodemographic and clinical factors and SRH outcomes.

**Results:**

Miscarriage and unsuccessful conception attempts were relatively high in our sample. Higher male partnered refugee age and lack of pregnancy desire were significant risk factors for miscarriage (OR:1.058; CI: 1.008– 1.110 and OR:5.190; CI: 1.031 – 26.112 respectively), while frequent antenatal care visits were protective (OR:0.923; CI: 0.855 – 0.997). Miscarriage was not associated with women’s age, and education. Unsuccessful conception attempts were significantly associated with higher male partnered refugee age (OR: 1.086; CI: 1.031 – 1.144), higher maternal age at first pregnancy (OR: 1.345; CI: 1.063 – 1.725), and lower number of alive children (OR: 0.436; CI: 0.313 – 0.607). No significant associations were found between unsuccessful conception attempts and screening, education, employment, and financial/transportation barriers to seeking healthcare services.

**Conclusion:**

Findings highlight the need for community-based interventions to address modifiable risk factors for miscarriage and unsuccessful conception among female Syrian refugees as described in detailed in the manuscript. This may include strengthening access to antenatal care, promoting family planning and optimal communication strategies, and addressing social and environmental determinants to mitigate related risk factors. Additionally, efforts should focus on improving SRH literacy and facilitating professional healthcare consultations and screenings.

**Supplementary Information:**

The online version contains supplementary material available at 10.1186/s12884-025-07990-6.

## Introduction

Global displacement crises have escalated to unprecedented levels, with an estimated 43.4 million refugees worldwide [1]. Syrian refugees constitute 6.4 million of these refugee populations, with approximately 1.5 million residing in Lebanon [2, 3], and 52% of them being women and girls. Syrian refugees encounter a variety of distinct socio-structural vulnerabilities associated with their displacement status that impact reproductive health outcomes. These include high poverty rates, systemic stressors, challenges with legal registration, substandard living conditions, and limited access to essential services, particularly sexual and reproductive health (SRH) services [4–6]. These factors combined, among others, pose significant risks on Female Syrian refugees (15- 24 year old), and increase their odds for poor SRH outcomes including maternal mortality. For example, refugee women and girls are at higher risk of experiencing complications during pregnancy and childbirth [5–8] as compared to host communities [9–11])and they are less likely to receive related health services when needed.

Abortion, both induced and spontaneous, stands as an important factor contributing to maternal mortality [12, 13]. It is estimated that approximately 73 million induced abortions occur globally each year [14]. Spontaneous abortion, also known as miscarriage [15], is the loss of a fetus prior to 20 weeks of gestation [16]. This adverse outcome affects 12–15% of pregnant women [17, 18], with refugee population being at particularly high risk [19]. For Syrians, induced abortion, is religiously forbidden and is illegal except in very limited conditions [5, 20, 21]. Miscarriages are increasing among Syrian refugee women following displacement [7] although in varying rates across host countries (10.5%, 15% and 23.1% in Lebanon, Jordan, and Turkey respectively) [15, 22, 23]. Several factors, including age, number of children, economic level, educational level, child marriage, and inadequate prenatal care have been showed to be associated with higher abortion and miscarriage rates [15, 24–28].

Another significant reproductive health problem linked to refugee status is infertility [29, 30]. As per the World Health Organization (WHO), infertility is the inability to achieve pregnancy after a year or more of regular, unprotected sexual intercourse” [29]. Globally, infertility affects around 70–80 million couples [31, 32]. While some couples face absolute fertility, the majority experience relative subfertility, whereby their opportunities to conceive are reduced [33]. A range of risk factors are linked to infertility, including high stress exposure, substandard living conditions, exposure to pollutants and toxins, and consanguineous marriage [30, 34–38]. In this study, we focus on unsuccessful conception attempts following the same aforementioned definition of infertility through self-report rather than clinical assessment.

Despite the reported prevalence rates of miscarriage and unsuccessful conception attempts among Syrian refugees in Lebanon, associated risk and protective factors among female Syrian refugees [12, 13, 39] have still not been fully examined. This study seeks to identify risk and protective for miscarriage and unsuccessful conception attempts among female Syrian refugees in Lebanon. By identifying these associations, we guide policies and interventions that aim to improve SRH outcomes among this marginalized population.

## Methodology

### SEEK project

This study is part of a broader project, the Self-Efficacy and Knowledge (SEEK) trial (protocol paper and retrospective trial registration under review at the time of submission). The purpose of this trial was to enhance Family Planning (FP) use, SRH outcomes, and wellbeing in humanitarian settings by developing, implementing, and evaluating the effect of a WHO low-intensity/low-resource integrated intervention package on SRH, family planning (FP), and mental health (MH). SEEK targeted refugee women and girls aged 15 to 24 who were, at the time of recruitment, married, not pregnant, and did not have any self-reported chronic condition.

SEEK study was conducted in the Bekaa region, which is located in eastern Lebanon along the border with Syria, and is predominantly a rural area that hosts the highest number of Syrian refugees [40]. Two primary health care centers (PHCs) met the project’s eligibility criteria. This cross-sectional study uses data focusing on miscarriage and unsuccessful conception attempts as outcomes. Miscarriage in this study was defined as spontaneous loss before 20 weeks gestation, and unsuccessful conception is defined as attempts at conception over 12-month period with no success. All data was collected through participants’ self-reporting through surveys, and no clinical diagnosis was obtained. Participants were first identified through the list of PHC patients by PHC staff, then called to participate in the study using an invitation script by the study’s research assistants. Data collection took place at the two PHCs.

Details regarding the sampling approach, eligibility criteria, data collection tools, and data collection procedures have been reported elsewhere (paper currently under review), however they may be accessible as supplementary material attached with this manuscript. The main survey used to collect data – the PAPFAM—has already been used in different studies and published elsewhere [41].

### Sampling strategy

In this study, a baseline prevalence of 30% of family planning use was derived from the estimated prevalence in Lebanon (34.5%) [42–44]. The required sample size was determined to be 380 adolescent girls and young women to detect a statistically significant change by 20% (to 50% family planning use) from a baseline rate of 30%. This change is estimated to have 90% statistical power at a significant level of p = 0.05, while allowing for a 30% attrition rate. To achieve this, eligibility screening was performed for 536 female Syrian refugees. Of those screened, 485 were found eligible and were subsequently requested to complete a baseline questionnaire. These 485 participants represent the entire eligible population identified through the screening process; no further selection or sub-sampling was performed from this eligible pool.

### Statistical analysis

Data was analyzed using State SE. Descriptive statistics were computed to summarize all study variables. For categorical variables, frequencies and percentages were reported. For continuous variables, means and standard deviations (SD) were calculated. The two primary outcomes were (1) history of miscarriage, and (2) unsuccessful attempts at conception, both of which were coded as binary variables (yes or no). Independent variables included sociodemographic characteristics and SRH-related experiences.

Bivariate analyses were conducted to explore associations between independent variables and outcomes of interest. Chi-square tests were used to categorical variables, while independent sample t-tests or one-way ANOVA were used for continuous variables. Variables that were statistically significant in the bivariate analyses (*p* < 0.05), along with age and education level, were entered into multivariable logistic regression models to assess their association with each outcome. Adjusted odds ratios (aOR) with 95% confidence intervals (CI) were reported. Statistical significance was set at *p* < 0.05.

### Ethical considerations

Ethical approval was sought and obtained from the Institutional Review Board at the American University of Beirut. All participants were required to provide written informed consent to participate, and for those under 18, their parents/caregivers were also required to provide informed consent on their behalf. Moreover, a referral list of Non-Governmental Organizations (NGOs) providing free mental health services was distributed to adolescent and young women who displayed symptoms of distress.

## Results

Descriptive results on the sociodemographic and clinical characteristics of the present sample have been reported elsewhere however part of them, as relevant to this study, can be found in Table 1 which shows demographics, pregnancy, and reproductive history of study participants (*n* = 485). Overall, the mean age of participants was 20.7 (SD = 2.3) with a range of 15 to 24, and their partners is 26.0 (SD = 2.0) with a range of 17 to 58. As for marriage age, it ranged from 12 to 24 with an average of 16.4 (SD = 2.0).

**Table 1:** Demographic, prenancy, and reproductive history of study participants (485) taken from Naal et al. [45] which is under review at the time of submission of this manuscript

	N (%) or Mean ± Std	
Demographics and Family Info		
Mean Age of		
Female partnered refugee	20.7	± 2.3
Male partnered refugee	26.0	± 5.3
Marriage (female partnered refugee)	16.4	± 2.0
Education of		
Female partnered refugee		
* Illiterate*	60	(12.4)
* Intermediate and below*	396	(81.8)
* Secondary and above*	28	(5.8)
Male partnered refugee		
* Illiterate*	81	(18.7)
* Intermediate and below*	319	(72.3)
* Secondary and above*	41	(9.3)
Working Status of		
Female partnered refugee		
* Currently Working (Yes)*	75	(15.46)
Male partnered refugee		
* Currently Working (Yes)*	369	(76.1)
Mean Number of alive children	1.9	± 1.1
Existing Kinship between the Couples (*Yes)*	248	(51.1)
Obstetric and Reproductive History		
Ever Pregnant *(Yes)*	393	(81)
Pregnancy desire of the woman when she was pregnant		
* She wanted to get pregnant at that time*	277	(70.5)
* She wanted to wait or not to get children at all*	101	(25.7)
Abortion or Miscarriage (*Yes)*	190	(39.2)
Infertility *(yes)*	136	(28)
Mean Age of the woman at First Pregnancy (Years)	17.2	± 2.0
Pregnancy screening (Yes)	374	(95.2)
Mean frequency of screening	5.0	± 3.3
Mean Month of 1 st check-up	2.0	± 1.5
Place of last Check-up		
* Hospital (private or public)*	16	(4.3)
* Health center/Clinic (private or public)*	336	(89.8)
* At Home*	22	(5.9)
Source of Consultation to become Pregnant *(yes)*	148	(30.5)
*Doctor*	42	(28.3)
*Relative*	85	(57.4)
*Both*	21	(14.2)
Barriers to Healthcare		
* Financial barrier (Yes)*	106	(21.9)
* Transportation Barrier (Yes)*	202	(41.7)

Table 2 displays the bivariate associations between socio-demographic and reproductive variables with the two outcomes: miscarriage and unsuccessful conception attempt. For unsuccessful conception attempt, significant associations were observed with the participants’ working status, where women working outside the home reported higher unsuccessful conception attempt rates compared to those not working (*p* = 0.012). In addition, unsuccessful conception attempt was significantly associated with higher age at marriage (mean = 16.96(SD = 2) vs. 16.12(SD = 2) years; *p* = 0.003), older age at first pregnancy (mean = 18.01(SD = 2.10) vs. 16.91 (SD = 1.98) years; *p* < 0.005), and fewer living children (mean = 1.25(SD = 0.97) vs. 2.03(SD = 1.09); *p* < 0.05). Women with no source of consultation reported significantly more unsuccessful conception attempt (p < 0.05). Transportation barriers showed a marginal association with unsuccessful conception attempt (*p* = 0.059), with higher rates reported among those experiencing such challenges.

**Table 2:** Bivariate association of the clinical reproductive health outcomes (Miscarriage and unsuccessful conception Attempt) with socio-demographics, obstetric and Reproductive History

	Miscarriage			unsuccessful conception Attempt		
	No (SD)	Yes (SD)	Sig	No (SD)	Yes (SD)	Sig
female partnered refugee Age	20.627 ± 2.39	20.93 ± 2.34	0.160	20.72 ± 2.47	20.8 ± 2.11	0.759
Male partnered refugee Age	25.35 ± 4.62	27.03 ± 5.96	0.001*	25.90 ± 4.52	26.30 ± 6.79	0.527
Marriage Age	16.60 ± 2.06	16.61 ± 1.96	0.025*	16. 2 ± 2	16.9 ± 2	0.003*
female partnered refugee Age at 1 st Pregnancy	17.24 ± 2.06	17.04 ± 1.89	0.315	16.91 ± 1.89	18.01 ± 2.10	< 0.05*
Education of female partnered refugee			0.254			0.143
*Illiterate*	32(10.88)	28(14.74)		41(11.78)	19(13.97)	
*Intermediate and below*	242(82.31)	154(81.05)		291(83.62)	105(77.21)	
*Secondary and above*	20(6.8)	8(4.21)		16(4.60)	12(8.82)	
Education of male partnered refugee			0.879			0.912
*Illiterate*	48(18.25)	33918.54)		60(18.81)	21(17.21)	
*Intermediate and below*	192(73)	127(71.35)		229(71.79)	90(73.77)	
*Secondary and above*	23(8.75)	18(10.11)		30(9.40)	11(9.02)	
Working Status of female partnered refugee			0.352			0.012*
*Do not practice any work*	253(85.76)	157(85.63)		304(87.11)	106(77.94)	
*Work outside the Home*	42(14.42)	33(17.37)		45(12.89)	30(22.06)	
Working Status male partnered refugee			0.302			0.065
*Unemployed*	48(16.27)	41(21.58)		73(20.92)	16(11.76)	
*Employed*	229(77.63)	140(73.68)		257(73.64)	112(82.53)	
*Other*	18(6.10)	9(4.74)		19(5.44)	5(5.88)	
Existing Kinship			0.688			0.270
*No*	142(48.14)	95(50)		176(50.43)	61(44.85)	
*Yes*	153(51.86)	95(50)		173(49.57)	75(55.15)	
Financial Barriers			0.410			0.160
*No*	181(61.65)	124(65.26)		226(64.94)	79 (58.09)	
*Yes*	113(38.44)	66(34.74)		112(35.06)	57 (41.91)	
Transportation Barriers			0.499			0.059
*Yes*	65(22.03)	37(19.47)		81(23.21)	21(15.44)	
*No*	230(77.79)	153(80.53)		268(76.79)	115(84.64)	
Number of Alive Children	1.89 ± 0.98	1.82 ± 1.25	0.534	2.03 ± 1.09	1.25 ± 0.97	< 0.05*
Frequency of Screening	5.34 ± 3.21	4.5 ± 3.36	0.016*	4.94 ± 3.19	5.02 ± 3.66	0.852
1 st Checkup during Pregnancy	2.10 ± 1.53	1.82 ± 1.25	0.133	2.0 ± 1.44	2.09 ± 1.51	0.570
Place of checkup			0.861			0.381
*Hospital*	9(4.33)	7(4.22)		10(3.44)	6(7.23)	
*Health center/Clinic*	188(90.38)	148(89.16)		264(90.72)	72(86.75)	
*Home*	11(5.29)	11 (6.63)		17(5.84)	5(6.02)	
Previous Pregnancy			< 0.05*			< 0.05 *
*No*	84(28.47)	8(4.21)		43(12.32)	49(36.03)	
*Yes*	211(71.53)	182(95.79)		306(87.68)	87(63.67)	
Pregnancy Desire			0.060			0.041*
* She wanted to get pregnant*	157(74.41)	120(65.93)		208(67.97)	69(79.31)	
* She wanted to wait*	48(22.75)	53(29.13)		85(27.78)	16(18.39)	

For miscarriage, significant associations were found with older male partner refugee age (mean = 27.03(SD = 5.96) vs. 25.35(SD = 4.62) years; *p* = 0.001), higher age at marriage of the female partnered refugee (mean = 16.61(SD = 1.96) vs. 16.00(SD = 2.06) years; p = 0.025), and greater number of previous pregnancies. Additionally, more frequent reproductive health screenings were significantly associated with miscarriage (*p* = 0.016). No significant associations were found for education, financial barriers, or source of consultation in this outcome group.

Male partner refugee age was positively associated with the likelihood of miscarriage; every year increase was associated with 5% increase in the odds of miscarriage (OR = 1.058;95% CI: 1.008–1.110; *p* = 0.023). Women who did not want to get pregnant at all had significantly higher odds of experiencing miscarriage (OR = 5.190, 95% CI: 1.031–26.112; *p* = 0.046) compared to those who wanted to get pregnant. Additionally, increased frequency of screening was associated with 8% decrease in the odds of miscarriage (OR = 0.923, 95% CI: 0.855–0.997; *p* = 0.041) (Table 3).

**Table 3:** Multivariable logistic regression for the Miscarriage outcome (Ref = No)

		Miscarriage	
	OR	(CI)	Sig
Female partnered refugee Age	1.062	(0.965 1.169)	0.216
Male partnered refugee Age	1.058	(1.008 1.110)	0.023*
Marriage Age	0.936	(0.823 1.064)	0.309
Education of female partnered refugee			
*Illiterate*	-	-	-
*Intermediate and below*	0.601	(0.294 1.227)	0.162
*Secondary and above*	0.588	(0.180 1.924)	0.380
Education of male partnered refugee			
*Illiterate*	-	-	-
*Intermediate and below*	0.934	(0.504 1.732)	0.828
*Secondary and above*	0.842	(0.329 2.156)	0.720
Pregnancy Desire			
*She wanted to get pregnant*	-	-	-
*She wanted to wait a little bit*	1.311	(0.772 2.226)	0.316
She didn’t want to get pregnant at all	5.190	(1.031 26.112)	0.046*
Financial Barriers			
*No*	-	-	-
*Yes*	1.037	(0.646 1.665)	0.881
Frequency of Screening	0.923	(0.855 0.997)	0.041*

As shown in Table 4, male partnered refugee age and the female partnered refugee age at her first pregnancy had a significant association with unsuccessful conception attempts: each one year increase in age was associated with 8% (OR = 1.086, 95% CI: 1.031–1.144; *p* = 0.002) and 35% increase in the odds of infertility respectively ((OR = 1.354, 95% CI: (0.063—1.725, *p* = 0.014). The odds of infertility was lower across women who have a higher number of alive children (OR = 0.436,CI: 0.313- 0.607, *p* = < 0.05).

**Table 4:** Multivariable logistic regressions for the unsuccessful conception attempts (Ref- = no)

	Infertility			
	OR	(CI)		Sig
Female partnered refugee Age	1.019	(0.905	1.147)	0.76
Male partnered refugee Age	1.086	(1.031	1.144)	0.002*
Marriage Age	0.841	(0.665	1.064)	0.15
female partnered refugee Age at 1 st Pregnancy	1.354	(1.063	1.725)	0.014*
Education of female partnered refugee				
*Illiterate*	-	-		-
*Intermediate and below*	0.735	(0.318	1.698)	0.471
*Secondary and above*	0.755	(0.211	2.699)	0.665
Education of male partnered refugee				
*Illiterate*	-	-		-
*Intermediate and below*	0.966	(0.448	2.083)	0.93
*Secondary and above*	0.733	(0.225	2.388)	0.607
Working Status of female partnered refugee				
*Unemployed*	-	-		-
*Employed*	0.677	(0.293	1.567)	0.363
Number of Alive Children	0.436	(0.313	0.607)	< 0.05*
Pregnancy Desire				
*She wanted to get pregnant*	-	-		-
*She wanted to wait a little bit*	0.626	(0.315	1.242)	0.18

Descriptive data on the sociodemographic and clinical characteristics of the sample are available in a separate manuscript currently under review and may be accessed through the supplementary material.

## Discussion

This study investigated risk factors associated with miscarriage and unsuccessful conception attempts among Syrian adolescents and young women in Lebanon, which is an under researched vulnerable population. It is also among the first to examine reproductive health outcomes among this group, while also accounting for male partner-related characteristics and using adjusted multivariable regression models to account for potential confounders. Our findings provide valuable insights, which can inform policies and interventions to improve those two SRH-related outcomes among this vulnerable population.

### Miscarriage

Miscarriage rates among Syrian adolescent girls and young women refugees was 39.2%, notably higher than documented rates in other regional contexts, such as Jordan (15%) and Turkey (23.1%) [15, 22]. This elevated rate underscores the unique challenge encountered by female Syrian refugees in Lebanon possibly due to the compounded effects of multiple recent stressors including economic and political instability and a fragmented, largely privatized healthcare system reliant on out-of-pocket expenditures, impacting access to reproductive health services [46–48]. It is also possible that such stressors and limited access to needed services may be exacerbated by psychological stressors, anxiety, and trauma associated with their displacement status which may also contribute poorer SRH outcomes [49, 50]. Also, female Syrian refugees often face unique stressors associated with exploitation from informal labor arrangements commonly due to limited legal protection which may pose additional health risks affecting reproduction outcomes [15, 24]. That said, mental health services have been significantly improving over time for refugees in Lebanon, given the expanded work of local and international NGOs in this sector who support more robust referral pathways, and easier access and service delivery. Many of these services, especially those provided by actors such as Medecins Sans Frontiers (MSF), International Rescue Committee (IRC), Lebanese Family Planning Association (LFPA), and UNFP, to name a few, include SRH and FP services supported by referrals to mental health services when needed, or integrated psychosocial support for refugees.

In contrast, Syrian refugees in Turkey have access to public health services (including primary to tertiary care) free of charge, with costs covered by the Disaster and Emergency Management Authority [51]. This model is supported by universal health coverage and expanded infrastructure, such as field hospitals in refugee camps, contributing to high service utilization—over 90% of refugees in camps reportedly had access to health services [52, 53]. Jordan has also adopted a coordinated health response with UN agencies, Non-Governmental Organizations (NGOs), and government actors, receiving the region’s highest share of humanitarian funding for reproductive health and establishing 17 clinics and mobile teams to improve coverage and outreach [46, 54]. Meanwhile, although the United Nations High Commissioner for refugees (UNHCR) subsidizes certain services, refugees often face copayments [55], transportation barriers, and shortage of qualified staff, resulting in uneven ANC coverage [56]. In this context, Lebanon could benefit from adopting key features from Turkey’s approach, where universal health coverage has expanded access to essential maternal and child health services, and from Jordan’s coordinated reproductive health outreach, both of which have enhanced service delivery for refugee populations [52, 57].

Our study demonstrated a significant association between male partnered refugee age and risk of miscarriage, with each additional year in paternal age being correlated with a 5% increase in the likelihood of miscarriage. Given that the mean paternal age in our sample was 26 years(SD = 5.3), this association may not be attributed to common factors of advanced paternal age of the male partnered refugee (30 and above) reported in the literature, which point toward diminished testicular function, increased DNA fragmentation, and other biological changes as main predisposing factors for miscarriages [58–65]. The main predisposing factors instead, may likely to be related to lifestyle and environmental factors, such as stress and exposure to pollutants [66–69]. This is particularly relevant for Syrian refugees, who often reside in substandard living conditions and face structural disadvantages including poor access to employment, education and other basic necessities [70]. In Lebanon, many Syrian refugees face chronic stress associated with economic insecurity, and uncertain legal status [71–73]. Due to legal barriers to formal employment and the absence of legal protection, many Syrian refugees are pushed into informal labor [71, 74], most commonly in construction and agriculture. Such working conditions are often hazardous, with limited occupational safeguards, and high exposure to pollutants and chemicals [73, 75, 76]. These adverse exposures have been linked to impaired fertility and adverse pregnancy outcomes [77, 78]. Within this context, male refugees may experience a greater cumulative burden of environmental, occupational, and psychosocial stressors, which could contribute to the observed associations between increased paternal age and miscarriage.

In contrast, no association was detected between the female partnered refugee age and the likelihood of miscarriage. With a mean maternal age of 20 years(SD = 2.3), our sample may lack the age variation necessary to detect maternal age-related effects observed in other studies [15, 79]. Similarly, age at marriage was not significantly associated with the risk of miscarriage. The mean marriage age was 16.4 years(SD = 2), which aligns with findings from a study in India that indicated no adverse pregnancy outcomes for women marrying between ages 15 and 17 [80]. Marrying before the age of 14 may be associated with higher risks for miscarriage due to physical and biological immaturity [80–83], however this age group was not targeted in our current study. Also, education of female partnered refugee and male partnered refugee was not found to be significantly associated with risk of miscarriage, possibly because our sample generally represented a group with low education, with little education-related variations among participants.

Our findings revealed that women who did not desire pregnancy had significantly higher miscarriage odds, consistent with research linking unintended pregnancy to increased risk of abortions and miscarriage [84, 85]. Unintended pregnancies are often associated with unhealthy behaviors and elevated levels of stress and depression, which could lead to adverse pregnancy outcomes [42, 43, 86, 87].

Frequency of screening during pregnancy was inversely related to miscarriage risk, with more antenatal care (ANC) visits associated with an 8% decrease in the odds of miscarriage. Studies showed that suboptimal ANC visits are associated with increased risk of miscarriage, while attending at least four ANC visits is linked to healthier maternal behaviors and improved outcomes [44, 88, 89]. This aligns with our findings, especially that the mean number of ANC visits was approximately 5(SD = 3.3) during pregnancy. This highlights the importance of regular ANC visits, especially for refugees with limited healthcare access, in improving pregnancy outcomes [56, 90–93].

### Unsuccessful conception attempts

Frequency of screening was not found to be associated with unsuccessful conception attempts, possibly highlighting that other factors may play a more important role. Generally, most of those who sought consultations did so from non-professional sources such as relatives and friends. Adolescent refugees often rely on relatives for information about their SRH [5, 94], and it is unsurprising that consultations from relatives, as opposed to health professionals, are more prevalent.

Current female partnered refugee’s age, pregnancy desire, educational level, transportation and financial barriers, and women’s employment status were not significantly associated with unsuccessful conception attempts. The relatively young age of women and men in our sample suggests the absence of age-related infertility risk, as such biological variables that may become more pronounced with advancing age, as reported in the literature [58, 95–99]. Additionally, the non-significant association between marriage age (mean age at marriage 16.4 years) and unsuccessful conception attempts is consistent with a study conducted in Turkey, which showed no link between marriage before age 29 and infertility [97]. This may be explained by the high fertility rates commonly observed in early marriages [100] given young ages.

Furthermore, the absence of an association between pregnancy desire, educational level, employment status, financial and transportation barriers, and unsuccessful conception attempts may suggest a limited direct role for these factors in fertility outcomes within our specific cohort. However, it is imperative to consider several contextual and methodological issues. For instance, the restricted variability in education attainment within our sample may have attenuated our statistical power to detect socioeconomic effects. Second, our measures may have no adequately captured nuanced stressors or environmental exposures that may be linked to altered fertility in prior research [101, 102]. Indeed, a growing body of literature demonstrates that social determinants, including low socioeconomic status and chronic psychological stress may exert profound physiological effects such as disrupting endocrine function or affecting ovarian reserve [103–107]. Therefore, our null findings may reflect measurement limitations or sample size restrictions. To clarify these complex relationships, future research should more clearly assess social determinants and environmental exposures in relation to clinical fertility.

Similar to above-mentioned risk factors for miscarriage, higher male partnered refugee age was found to be significantly associated with unsuccessful conception attempts. Also, higher female partnered refugee’s age at first pregnancy, and lower number of alive children were also found to be significantly associated with unsuccessful conception attempts.

### Limitations

This study has several limitations. First, a causal relationship between the detected risk factors and the reproductive health outcomes could not be established due to the cross-sectional design used. This design also presented some limitations because no clinical diagnoses were conducted to confirm medical conditions such as miscarriage or infertility for example which may introduce misclassification bias. Second, the target population includes solely Syrian adolescent girls and young women refugees residing in the Bekaa governorate which may limit external validity of the findings. Data was collected from two PHCs in the Bekaa because of logistical feasibility, and because this region accommodates the largest proportion of refugees, and they tend to be homogenous when compared to Syrian refugees living in other areas in Lebanon with regard to social and cultural characteristics [40].

Moreover, there was little variation in key demographic characteristics, including age and educational levels. Displacement status and duration were also not explored, and these could be important factors to consider in this study. This might affect the ability to identify potentially significant associations between some variables in relation to SRH outcomes. Furthermore, in a sensitive topic like SRH, reliance on self-reported data may be subject to social desirability bias, potentially affecting the accuracy of information. Participants may underreport miscarriage and unsuccessful conception experiences due to stigma, however this was partially mitigated by assigning female data collectors from the same communities of participants, supported by local women community members to promote trust good relationships.

Lastly, this study did not account for some variables that may influence reproductive outcomes, such as the quality of ANC services or underlying medical conditions. Further research is required to understand the broader social, cultural, and health system factors influencing reproductive health among refugee populations.

### Conclusion & implications

This study explored risk and protective factors associated with SRH of female Syrian refugees in Lebanon, particularly regarding miscarriage and unsuccessful conception attempts. Our findings showed that higher age of male partnered refugee and no desire for pregnancy, were significant risk factors for miscarriages, whereas higher frequency of screening was a protective factor. As for unsuccessful conception, our findings showed that higher male partnered refugee age and higher female partnered refugee age at first pregnancy were significant risk factors, whereas higher number of alive children was a protective factor. Revised evidence-based policies and community-based interventions are needed to target key modifiable risk factors.

Given the characteristics and living conditions of the present sample, community-based interventions may be best fit, in combination with facilitating access to affordable and accessible healthcare services.

Specifically, to address negative outcomes associated with miscarriages, interventions may focus on working with women on family planning strategies and optimal communication approaches to support those who have no desire of getting pregnant to better navigate stressors and pressures from their communities and husbands who may want another child. Our results show that women who have no desire to get pregnant have increased risk of miscarriage, and when combined with other stressors, this may result in detrimental health outcomes. Also, it is important to support pregnant women to seek additional periodic screening since our results showed that this may be a protective factor as it decreases the odds of miscarriages.

Based on our results, social and environmental risk factors may also need to be targeted in community-based interventions, especially for men of higher age, since this was shown to be a risk factor for both miscarriages and unsuccessful conception attempts. However, a deeper analysis into the relationship between social and environmental factors and SRH outcomes may be needed by future research to better highlight risk and protective factors and inform interventions.

Finally, for unsuccessful conception attempts, it is important to target women of higher age, and who have lower number of alive children. This may include a combination of educational and awareness raising sessions about fertility to increase odds of desired conception, improving access to consultations from healthcare workers to motivate them to seek professional help as opposed to peer advice, and tailoring mental health and psychosocial support since repeated unsuccessful pregnancies may have important mental health impacts on women.

## Supplementary Information

Supplementary Material 1.

## Data Availability

The datasets used and/or analyzed during the current study are available from the corresponding author on reasonable request.

## References

[1] UNHCR. Global Trends Forced Displacement in 2023 2023 [Available from: https://www.unhcr.org/global-trends-report-2023.

[2] UNHCR. Lebanon 2024 [Available from: https://reporting.unhcr.org/operational/operations/lebanon.

[3] UNHCR. DATA AND STATISTICS Global Trends. 2023.

[4] UNHCR. UNHCR Lebanon at a Glance 2024 [Available from: https://www.unhcr.org/lb/at-a-glance.

[5] Fahme SA, Sieverding M, Abdulrahim S. Sexual and reproductive health of adolescent Syrian refugee girls in Lebanon: a qualitative study of healthcare provider and educator perspectives. Reprod Health. 2021;18(1):113. 34092236 10.1186/s12978-021-01170-3 PMC8183084

[6] UN_Women. Follow-up assessment on gendered realities in displacement: Lebanon. n.d.

[7] AlArab N, Nabulsi D, El Arnaout N, Dimassi H, Harb R, Lahoud J, et al. Reproductive health of Syrian refugee women in Lebanon: a descriptive analysis of the Sijilli electronic health records database. BMC Womens Health. 2023;23(1):81. 36823589 10.1186/s12905-023-02231-4 PMC9951425

[8] Abdulrahim S, DeJong J, Mourtada R, Zurayk H. Estimates of early marriage among Syrian refugees in Lebanon in 2016 compared to Syria pre-2011: Sawsan Abdulrahim. Eur J Public Health. 2017;27(suppl_3):ckx189. 049.

[9] Gissler M, Alexander S, MacFarlane A, Small R, Stray-Pedersen B, Zeitlin J, et al. Stillbirths and infant deaths among migrants in industrialized countries. Acta Obstet Gynecol Scand. 2009;88(2):134–48. 19096947 10.1080/00016340802603805

[10] Liu C, Ahlberg M, Hjern A, Stephansson O. Perinatal health of refugee and asylum-seeking women in Sweden 2014–17: a register-based cohort study. Eur J Public Health. 2019;29(6):1048–55. 31274154 10.1093/eurpub/ckz120 PMC6896976

[11] UNFPA. As Syrian crisis drags into fifth year, pregnant women caught in the middle2015. Available from: https://www.unfpa.org/news/syrian-crisis-drags-fifth-year-pregnant-women-caught-middle.

[12] Kassebaum NJ, Barber RM, Bhutta ZA, Dandona L, Gething PW, Hay SI, et al. Global, regional, and national levels of maternal mortality, 1990–2015: a systematic analysis for the Global Burden of Disease Study 2015. The lancet. 2016;388(10053):1775–812. 10.1016/S0140-6736(16)31470-2 PMC5224694 27733286

[13] Dönmez A, Çoban A, Canbay F. Unwanted pregnancy and unsafe abortions of solution the role midwife's. 2016.

[14] WHO. Abortion 2024. Available from: https://www.who.int/news-room/fact-sheets/detail/abortion.

[15] Khadra MM, Suradi HH, Amarin JZ, El-Bassel N, Kaushal N, Jaber RM, et al. Risk factors for miscarriage in Syrian refugee women living in non-camp settings in Jordan: results from the women ASPIRE cross-sectional study. Conflict Health. 2022;16(1):32. 35672855 10.1186/s13031-022-00464-y PMC9171994

[16] Frederiksen LE, Ernst A, Brix N, Lauridsen LLB, Roos L, Ramlau-Hansen CH, et al. Risk of adverse pregnancy outcomes at advanced maternal age. Obstet Gynecol. 2018;131(3):457–63. 29420406 10.1097/AOG.0000000000002504

[17] Sedgh G, Singh S, Hussain R. Intended and unintended pregnancies worldwide in 2012 and recent trends. Stud Fam Plann. 2014;45(3):301–14. 25207494 10.1111/j.1728-4465.2014.00393.x PMC4727534

[18] Magnus MC, Wilcox AJ, Morken NH, Weinberg CR, Håberg SE. Role of maternal age and pregnancy history in risk of miscarriage: prospective register based study. bmj. 2019;364. 10.1136/bmj.l869 PMC6425455 30894356

[19] Harakow HI, Hvidman L, Wejse C, Eiset AH. Pregnancy complications among refugee women: a systematic review. Acta Obstet Gynecol Scand. 2021;100(4):649–57. 33372265 10.1111/aogs.14070

[20] Cherri Z, Gil Cuesta J, Rodriguez-Llanes JM, Guha-Sapir D. Early marriage and barriers to contraception among Syrian refugee women in Lebanon: a qualitative study. Int J Environ Res Public Health. 2017;14(8):836. 28757595 10.3390/ijerph14080836 PMC5580540

[21] Bashour H, Abdulsalam A, Jabr A, Cheikha S, Tabbaa M, Lahham M, et al. Maternal mortality in Syria: causes, contributing factors and preventability. Trop Med Int Health. 2009;14(9):1122–7. 19624475 10.1111/j.1365-3156.2009.02343.x

[22] Torun P, Karaaslan MücazM, Sandıklı B, Acar C, Shurtleff E, Dhrolia S, et al. Health and health care access for Syrian refugees living in İstanbul. Int J Public Health. 2018;63(5):601–8. 29629476 10.1007/s00038-018-1096-4

[23] Usta J, Masterson AR. Women and health in refugee settings: the case of displaced Syrian women in Lebanon. Gender-based violence: Perspectives from Africa, the Middle East, and India. 2015:119–43.

[24] Al-Alami Z, Abu-Huwaij R, Hamadneh S, Taybeh E. Understanding miscarriage prevalence and risk factors: insights from women in Jordan. Medicina (B Aires). 2024;60(7):1044. 10.3390/medicina60071044 PMC11279235 39064473

[25] Megersa BS, Ojengbede OA, Deckert A, Fawole OI. Factors associated with induced abortion among women of reproductive age attending selected health facilities in Addis Ababa, Ethiopia: a case control study. BMC Womens Health. 2020;20(1):188. 32883263 10.1186/s12905-020-01023-4 PMC7469090

[26] Yeboah I, Okyere J, Klu D, Agbadi P, Agyekum MW. Individual and community-level factors associated with repeat induced abortion among women in Ghana: a multivariable complex sample logistic regression analysis of 2017 Ghana maternal health survey. BMC Public Health. 2024;24(1): 1420. 38807108 10.1186/s12889-024-18948-2 PMC11131185

[27] Chae S, Desai S, Crowell M, Sedgh G, Singh S. Characteristics of women obtaining induced abortions in selected low-and middle-income countries. PLoS ONE. 2017;12(3):e0172976. 28355285 10.1371/journal.pone.0172976 PMC5371299

[28] Danso NA, Nagumsi B, Twi-Yeboah A, Boamah F, Cadri A. The rate of abortion in Ghana among women of reproductive age: analysis of the 2007 and 2017 maternal health surveys. Ethics Med Public Health. 2022;22:100769.

[29] WHO. Infertility. 2024. Available from: https://www.who.int/news-room/fact-sheets/detail/infertility.

[30] Dhair A, Abed Y. The association of types, intensities and frequencies of physical activity with primary infertility among females in Gaza Strip, Palestine: a case-control study. PLoS ONE. 2020;15(10):e0241043. 33095804 10.1371/journal.pone.0241043 PMC7584224

[31] Lindsay TJ, Vitrikas KR. Evaluation and treatment of infertility. Am Fam Physician. 2015;91(5):308–14. 25822387

[32] Ho JR, Hoffman JR, Aghajanova L, Smith JF, Cardenas M, Herndon CN. Demographic analysis of a low resource, socioculturally diverse urban community presenting for infertility care in a United States public hospital. Contracept Reprod Med. 2017;2: 17. 29201422 10.1186/s40834-017-0044-7 PMC5683225

[33] Taylor A. Extent of the problem. BMJ. 2003;327(7412):434–6. 12933733 10.1136/bmj.327.7412.434 PMC188498

[34] Inhorn MC. Searching for love and test-tube babies: Iraqi refugee men in reproductive exile on the margins of Detroit. Med Anthropol. 2018;37(2):145–57. 28059554 10.1080/01459740.2016.1276904

[35] Inhorn MC, Birenbaum-Carmeli D, Tremayne S, Gürtin ZB. Assisted reproduction and Middle East kinship: a regional and religious comparison. Reprod Biomed Soc Online. 2017;4:41–51. 29774265 10.1016/j.rbms.2017.06.003 PMC5952653

[36] Nachtigall RD. International disparities in access to infertility services. Fertil Steril. 2006;85(4):871–5. 16580367 10.1016/j.fertnstert.2005.08.066

[37] Gore AC, Chappell VA, Fenton SE, Flaws JA, Nadal A, Prins GS, et al. EDC-2: the Endocrine Society’s second scientific statement on endocrine-disrupting chemicals. Endocr Rev. 2015;36(6):E1–150. 26544531 10.1210/er.2015-1010 PMC4702494

[38] Segal TR, Giudice LC. Before the beginning: environmental exposures and reproductive and obstetrical outcomes. Fertil Steril. 2019;112(4):613–21. 31561863 10.1016/j.fertnstert.2019.08.001

[39] Dhair A, Abed Y. The effect of preconception housing and living conditions on primary infertility among couples in Gaza Strip, Palestine: a case-control study. J Health Soc Sci. 2020;5:355–68.

[40] UNHCR. Operational Data Portal. 2023.

[41] Kobeissi L, El Kak FH, Khawaja M, Khoshnood K. HIV/AIDS-related knowledge and its association with socioeconomic status among women: results of Lebanese Survey for Family Health (PAPFAM) 2004. Asia Pac J Public Health. 2015;27(2):Np734–45. 10.1177/1010539511431299 22186399

[42] Ranatunga IDJC, Jayaratne K. Proportion of unplanned pregnancies, their determinants and health outcomes of women delivering at a teaching hospital in Sri Lanka. BMC Pregnancy Childbirth. 2020;20(1):667. 33153469 10.1186/s12884-020-03259-2 PMC7643445

[43] Surita FG, Paulino DSM, Pinho-Pompeu M. Health-related behaviors in pregnancy: a key to achieve better outcomes. Rev Bras Ginecol Obstet. 2020;42(3):121–3. 32232818 10.1055/s-0040-1708094 PMC10316843

[44] Kuhnt J, Vollmer S. Antenatal care services and its implications for vital and health outcomes of children: evidence from 193 surveys in 69 low-income and middle-income countries. BMJ Open. 2017;7(11):e017122. 29146636 10.1136/bmjopen-2017-017122 PMC5695442

[45] Naal H, El Dakdouki A, Sarieddine D, Abou-Honein Haidar G, Chamseddine Z, Fouad F, Bosqui T, El Iskandarani K, Tamim H, Ibrahim S, Abdul Sater Z, Saleh S. Characteristics of Family Planning and Sexual Reproductive Health among Syrian Refugee Women and Girls in Lebanon. Under Review BMC Women’s Health.

[46] Mhlongo S, Mason-Jones AJ, Ford K. Sexual, reproductive and mental health among young men (10–24) in low-and-middle income countries: a scoping review. Front Reprod Health. 2023;2023:5. 10.3389/frph.2023.1119407 PMC10725937 38111839

[47] Assi R, Özger-İlhan S, İlhan MN. Health needs and access to health care: the case of Syrian refugees in Turkey. Public Health. 2019;172:146–52. 31235210 10.1016/j.puhe.2019.05.004

[48] Şimşek Z, Yentur Doni N, Gül Hilali N, Yildirimkaya G. A community-based survey on Syrian refugee women’s health and its predictors in Şanliurfa. Turkey Women Health. 2018;58(6):617–31. 28430082 10.1080/03630242.2017.1321609

[49] Sahlool Z, Sankri-Tarbichi AG, Kherallah M. Evaluation report of health care services at the Syrian refugee camps in Turkey. Avicenna J Med. 2012;2(2):25–8. 23210017 10.4103/2231-0770.99148 PMC3507073

[50] UNHCR. Operational data portal-Health Working Group – Jordan. 2025. Available from: https://data.unhcr.org/en/working-group/48.

[51] Hemadeh R, Hammoud R, Kdouh O. Lebanon’s essential health care benefit package: a gateway for universal health coverage. Int J Health Plann Manage. 2019;34(4):e1921–36. 31271234 10.1002/hpm.2850

[52] Benage M, Greenough PG, Vinck P, Omeira N, Pham P. An assessment of antenatal care among Syrian refugees in Lebanon. Confl Health. 2015;9:8. 25741381 10.1186/s13031-015-0035-8 PMC4349304

[53] UNHCR. Health sector humanitarian response strategy. 2015.

[54] Kaltsas A, Moustakli E, Zikopoulos A, Georgiou I, Dimitriadis F, Symeonidis EN, et al. Impact of advanced paternal age on fertility and risks of genetic disorders in offspring. Genes. 2023;14(2):486. 36833413 10.3390/genes14020486 PMC9957550

[55] Sampson N, Untergasser G, Plas E, Berger P. The ageing male reproductive tract. J Pathol. 2007;211(2):206–18. 17200938 10.1002/path.2077

[56] de La Rochebrochard E, Thonneau P. Paternal age: are the risks of infecundity and miscarriage higher when the man is aged 40 years or over? Rev Epidemiol Sante Publique. 2005;53:47–55. 16471144

[57] Bartolacci A, Pagliardini L, Makieva S, Salonia A, Papaleo E, Viganò P. Abnormal sperm concentration and motility as well as advanced paternal age compromise early embryonic development but not pregnancy outcomes: a retrospective study of 1266 ICSI cycles. J Assist Reprod Genet. 2018;35:1897–903. 29995229 10.1007/s10815-018-1256-8 PMC6150884

[58] Luna M, Finkler E, Barritt J, Bar-Chama N, Sandler B, Copperman AB, et al. Paternal age and assisted reproductive technology outcome in ovum recipients. Fertil Steril. 2009;92(5):1772–5. 19539905 10.1016/j.fertnstert.2009.05.036

[59] Gonzalez DC, Ory J, Blachman-Braun R, Nackeeran S, Best JC, Ramasamy R. Advanced paternal age and sperm DNA fragmentation: a systematic review. World J Mens Health. 2022;40(1):104. 33987998 10.5534/wjmh.200195 PMC8761235

[60] Kaltsas A, Zikopoulos A, Vrachnis D, Skentou C, Symeonidis EN, Dimitriadis F, et al. Advanced paternal age in focus: unraveling its influence on assisted reproductive technology outcomes. J Clin Med. 2024;13(10):2731. 38792276 10.3390/jcm13102731 PMC11122544

[61] Kaarouch I, Bouamoud N, Madkour A, Louanjli N, Saadani B, Assou S, et al. Paternal age: negative impact on sperm genome decays and IVF outcomes after 40 years. Mol Reprod Dev. 2018;85(3):271–80. 29392876 10.1002/mrd.22963

[62] Esteves SC, Santi D, Simoni M. An update on clinical and surgical interventions to reduce sperm DNA fragmentation in infertile men. Andrology. 2020;8(1):53–81. 31692293 10.1111/andr.12724

[63] Agarwal A, Majzoub A, Baskaran S, Panner Selvam MK, Cho CL, Henkel R, et al. Sperm DNA fragmentation: a new guideline for clinicians. World J Mens Health. 2020;38(4):412–71. 32777871 10.5534/wjmh.200128 PMC7502318

[64] Pearce KL, Hill A, Tremellen KP. Obesity related metabolic endotoxemia is associated with oxidative stress and impaired sperm DNA integrity. Basic Clin Androl. 2019;29:1–9. 31114691 10.1186/s12610-019-0087-5 PMC6513521

[65] Ilacqua A, Izzo G, Emerenziani GP, Baldari C, Aversa A. Lifestyle and fertility: the influence of stress and quality of life on male fertility. Reprod Biol Endocrinol. 2018;16(1): 115. 30474562 10.1186/s12958-018-0436-9 PMC6260894

[66] Alaouie M, Troisi GM. Biomonitoring environmental exposure in Syrian refugees in Lebanon. Epidemiologia. 2024;5(2):309–17. 38920756 10.3390/epidemiologia5020021 PMC11203224

[67] Krafft C, Malaeb B, Al Zoubi S. How do policy approaches affect refugee economic outcomes? Insights from studies of Syrian refugees in Jordan and Lebanon. Oxf Rev Econ Policy. 2022;38(3):654–77.

[68] Doctors_Without_Borders. Syrians in Lebanon fear seeking medical care amid rising anti-refugee sentiment. 2024.

[69] Leaders_Consortium. Dignity at Stake: Challenges to Accessing Decent Work in Lebanon. 2019.

[70] Ruhnke SA, Hertner L, Köhler J, Kluge U. Social ecological determinants of the mental distress among Syrian refugees in Lebanon and Turkey: a transnational perspective. Soc Sci Med. 2024;346:116700. 38430874 10.1016/j.socscimed.2024.116700

[71] Noory B, Habib RR, Nuwayhid I. Exposure of Syrian refugee agricultural workers to pesticides in Lebanon: a socio-economic and political lens. Front Public Health. 2024;12: 1402511. 38993703 10.3389/fpubh.2024.1402511 PMC11236552

[72] Habib RR, Mikati D, Hojeij S, El Asmar K, Chaaya M, Zurayk R. Associations between poor living conditions and multi-morbidity among Syrian migrant agricultural workers in Lebanon. Eur J Public Health. 2016;26(6):1039–44. 27402635 10.1093/eurpub/ckw096 PMC5172490

[73] Kumar N, Singh AK. Impact of environmental factors on human semen quality and male fertility: a narrative review. Environ Sci Eur. 2022;34(1):6.

[74] Bruner-Tran KL, Mokshagundam S, Barlow A, Ding T, Osteen KG. Paternal environmental toxicant exposure and risk of adverse pregnancy outcomes. Curr Obstet Gynecol Rep. 2019;8(3):103–13. 32953240 10.1007/s13669-019-00265-w PMC7500507

[75] Quenby S, Gallos ID, Dhillon-Smith RK, Podesek M, Stephenson MD, Fisher J, et al. Miscarriage matters: the epidemiological, physical, psychological, and economic costs of early pregnancy loss. Lancet. 2021;397(10285):1658–67. 33915094 10.1016/S0140-6736(21)00682-6

[76] Paul P. Maternal age at marriage and adverse pregnancy outcomes: findings from the India human development survey, 2011–2012. J Pediatr Adolesc Gynecol. 2018;31(6):620–4. 30107232 10.1016/j.jpag.2018.08.004

[77] Dixon-Mueller R. How young is “too young”? Comparative perspectives on adolescent sexual, marital, and reproductive transitions. Stud Fam Plann. 2008;39(4):247–62. 19248713 10.1111/j.1728-4465.2008.00173.x

[78] Ganchimeg T, Ota E, Morisaki N, Laopaiboon M, Lumbiganon P, Zhang J, et al. Pregnancy and childbirth outcomes among adolescent mothers: a World Health Organization multicountry study. BJOG. 2014;121(Suppl 1):40–8. 24641534 10.1111/1471-0528.12630

[79] Alam N. Teenage motherhood and infant mortality in Bangladesh: maternal age-dependent effect of parity one. J Biosoc Sci. 2000;32(2):229–36. 10765612 10.1017/s0021932000002297

[80] Yazdkhasti M, Pourreza A, Pirak A, Abdi F. Unintended pregnancy and its adverse social and economic consequences on health system: a narrative review article. Iran J Public Health. 2015;44(1):12–21. 26060771 PMC4449999

[81] Assefa N, Berhane Y, Worku A, Tsui A. The hazard of pregnancy loss and stillbirth among women in Kersa, East Ethiopia: a follow up study. Sex Reprod Healthc. 2012;3(3):107–12. 22980735 10.1016/j.srhc.2012.06.002

[82] McCrory C, McNally S. The effect of pregnancy intention on maternal prenatal behaviours and parent and child health: results of an Irish cohort study. Paediatr Perinat Epidemiol. 2013;27(2):208–15. 23374066 10.1111/ppe.12027

[83] Han JY, Nava-Ocampo AA, Koren G. Unintended pregnancies and exposure to potential human teratogens. Birth Defects Res A. 2005;73(4):245–8. 10.1002/bdra.20132 15786494

[84] Mourtada R, Melnikas AJ. Syrian refugee women’s access to family planning services and modern contraception during overlapping crises in Bekaa, Lebanon. BMC Womens Health. 2023;23(1):475. 37674178 10.1186/s12905-023-02613-8 PMC10481481

[85] WHO. Refugee and migrant mental health. 2025.

[86] Samari G. Syrian refugee women’s health in Lebanon, Turkey, and Jordan and recommendations for improved practice. World Med Health Policy. 2017;9(2):255–74. 29051840 10.1002/wmh3.231 PMC5642924

[87] Haftu A, Hagos H, Mehari MA, G/her B. Pregnant women adherence level to antenatal care visit and its effect on perinatal outcome among mothers in Tigray public health institutions, 2017: cohort study. BMC research notes. 2018;11:1–6. 10.1186/s13104-018-3987-0 PMC6286528 30526644

[88] Dimassi H, Alameddine M, Sabra N, El Arnaout N, Harb R, Hamadeh R, et al. Maternal health outcomes in the context of fragility: a retrospective study from Lebanon. Conflict Health. 2023;17(1):59. 38093261 10.1186/s13031-023-00558-1 PMC10720064

[89] Yeshialem E, Abera M, Tesfay A. Determinants of adverse pregnancy outcomes among mothers who gave birth from Jan 1-Dec 31/2015 in Jimma university specialized hospital, case control study, 2016. Ethiop J Reprod Health. 2019;11(1):10.

[90] Hailemichael HT, Debelew GT, Alema HB, Weldu MG, Misgina KH. Determinants of adverse birth outcome in Tigrai region, North Ethiopia: hospital-based case-control study. BMC Pediatr. 2020;20:1–9. 31914947 10.1186/s12887-019-1835-6 PMC6947822

[91] Worku AG, Yalew AW, Afework MF. The contributions of maternity care to reducing adverse pregnancy outcomes: a cohort study in Dabat district, Northwest Ethiopia. Matern Child Health J. 2014;18:1336–44. 24045911 10.1007/s10995-013-1367-x

[92] Woday A, Muluneh MD, Sherif S. Determinants of preterm birth among mothers who gave birth at public hospitals in the Amhara region, Ethiopia: a case-control study. PLoS ONE. 2019;14(11):e0225060. 31710645 10.1371/journal.pone.0225060 PMC6844458

[93] Korri R, Froeschl G, Ivanova O. A cross-sectional quantitative study on sexual and reproductive health knowledge and access to services of Arab and Kurdish Syrian refugee young women living in an urban setting in Lebanon. Int J Environ Res Public Health. 2021;18(18):9586. 34574511 10.3390/ijerph18189586 PMC8471977

[94] ASRM. Age Infertility in Women. 2006.

[95] Walker MH, Tobler KJ. Female infertility. 2020. 32310493

[96] Sarac M, Koc I. Prevalence and risk factors of infertility in Turkey: evidence from demographic and health surveys, 1993–2013. J Biosoc Sci. 2018;50(4):472–90. 28641583 10.1017/S0021932017000244

[97] Romero Ramos R, Romero Gutiérrez G, Abortes Monroy I, Medina Sánchez HG. Risk factors associated to female infertility. Ginecol Obstet Mex. 2008;76(12):717–21. 19149400

[98] Bushnik T, Cook JL, Yuzpe AA, Tough S, Collins J. Estimating the prevalence of infertility in Canada. Hum Reprod. 2012;27(3):738–46. 22258658 10.1093/humrep/der465 PMC3279129

[99] Raj A, Saggurti N, Balaiah D, Silverman JG. Prevalence of child marriage and its effect on fertility and fertility-control outcomes of young women in India: a cross-sectional, observational study. Lancet. 2009;373(9678):1883–9. 19278721 10.1016/S0140-6736(09)60246-4 PMC2759702

[100] Palomba S, Daolio J, Romeo S, Battaglia FA, Marci R, La Sala GB. Lifestyle and fertility: the influence of stress and quality of life on female fertility. Reprod Biol Endocrinol. 2018;16(1):113. 30501641 10.1186/s12958-018-0434-y PMC6275085

[101] Pizzorno J. Environmental toxins and infertility. Integr Med (Encinitas). 2018;17(2):8–11. 30962779 PMC6396757

[102] Barut MU, Agacayak E, Bozkurt M, Aksu T, Gul T. There is a positive correlation between socioeconomic status and ovarian reserve in women of reproductive age. Med Sci Monitor. 2016;22:4386. 10.12659/MSM.897620 PMC5123781 27847382

[103] Hu Y, Wang W, Ma W, Wang W, Ren W, Wang S, et al. Impact of psychological stress on ovarian function: Insights, mechanisms and intervention strategies (Review). Int J Mol Med. 2025;55(2). 10.3892/ijmm.2024.5475 PMC11670866 39704226

[104] Li XF, Mitchell JC, Wood S, Coen CW, Lightman SL, O’Byrne KT. The effect of oestradiol and progesterone on hypoglycaemic stress-induced suppression of pulsatile luteinizing hormone release and on corticotropin-releasing hormone mRNA expression in the rat. J Neuroendocrinol. 2003;15(5):468–76. 12694372 10.1046/j.1365-2826.2003.01014.x

[105] Ferin M. Neuropeptides, the stress response, and the hypothalamo-pituitary-gonadal axis in the female rhesus monkey. Ann N Y Acad Sci. 1993;697:106–16. 8257005 10.1111/j.1749-6632.1993.tb49927.x

[106] Breen KM, Karsch FJ. New insights regarding glucocorticoids, stress and gonadotropin suppression. Front Neuroendocrinol. 2006;27(2):233–45. 16712908 10.1016/j.yfrne.2006.03.335

[107] Devi S. Economic crisis hits Lebanese health care. Lancet. 2020;395(10224):548. 32087781 10.1016/S0140-6736(20)30407-4

