# Supplementary material for: Demographic and clinical associations with miscarriage and unsuccessful conception attempts among female Syrian refugees in Lebanon
Source: BMC Pregnancy Childbirth. 2025 Aug 12;25:837. doi: 10.1186/s12884-025-07990-6 (PMC12341242; doi:10.1186/s12884-025-07990-6)
Supplement: Supplementary file 1 — Supplementary Material 1. [file 12884_2025_7990_MOESM1_ESM.docx]

**Supplementary Material - Methodology**

**Eligibility criteria**

The target population was Syrian adolescent girls and young women refugees aged 15 to 24 years who had been living in Lebanon for at least 6 months and were willing to participate in the study. This population should also fulfill the following requirements: (1) married; (2) not currently breastfeeding; (3) not pregnant; (4) free from chronic conditions that might interfere with the main study adherence; (5) not currently receiving specialized mental health care; and (6) willing to participate in the project. Adolescent girls and young women who did not the eligibility criteria outlined above, were not included in the study.

***Procedures***

**Piloting**

The data collection tool was piloted before the data collection process. The aim of piloting was to ensure the accuracy and comprehensibility of the data collection tool. Five Syrian refugee women selected from our database established through multiple projects targeting this population were requested to participate in the piloting process. These women, who had demographic characteristics like the study’s target population, signed a consent form before filling out the survey. Following their feedback, the questionnaire was modified accordingly.

**Recruitment of Participants**

19 data collection days at the selected PHCs were conducted between November and December 2023. The aim was to recruit the target sample size for the study. Based on the eligibility criteria listed above, staff from each selected PHC reviewed their database and contacted Syrian young women over 18 years old and the legal guardian of those under 18 by phone and invited them to attend the PHC. During data collection, the invited adolescent and young women were rescreened for eligibility, and those who met the criteria and were willing to participate were asked to fill a baseline questionnaire. Each invitee received, as incentives, transportation fees, a women’s hygiene kit, and free optional gynecological consultations.

**Data collection**

A screening questionnaire was used to assess the eligibility of the participants. Eligibility screening was performed for 536 Syrian adolescent girls and young women refugees. Of whom, 485 were found eligible and were requested to complete a baseline questionnaire. Before the data collection process, participants aged 18 and older were requested to sign a consent form; participants who were under 18 signed an ascent form, and their parents signed a parental consent.

Acknowledging that women feel more comfortable discussing sexual topics with other women, female data collectors were assigned to the data collection process. These females were trained on ethical concerns, including privacy, confidentiality, potential risks to participants, informed consent, and techniques to maintain the accuracy of data collection. Furthermore, to maintain high confidentiality, the research team replaced the direct personal identifiers of participants with a unique, predefined identification code.

***Data collection tool***

The Arabic validated Pan Arab-Family Health (PAPAFAM) tool was used. This tool had several sections related to family health. For the purpose of our study, we selected the sections focusing on participants’ backgrounds, marriage, pregnancy, and reproduction (87).
